# Dysferlin mutations and mitochondrial dysfunction

**DOI:** 10.1016/j.nmd.2016.08.008

**Published:** 2016-11

**Authors:** Amy E. Vincent, Hannah S. Rosa, Charlotte L. Alston, John P. Grady, Karolina A. Rygiel, Mariana C. Rocha, Rita Barresi, Robert W. Taylor, Doug M. Turnbull

**Affiliations:** a Wellcome Trust Centre for Mitochondrial Research, Institute of Neuroscience, Newcastle University, Newcastle upon Tyne, NE2 4HH, UK; b Rare Diseases Advisory Group Service for Rare Neuromuscular Diseases, Muscle Immunoanalysis Unit, Newcastle-upon-Tyne Hospitals NHS Foundation Trust, Newcastle upon Tyne NE2 4AZ, UK

**Keywords:** Dysferlin, Mitochondria, LGMD2B, Immunofluorescence, Histochemistry, Cytochrome *c* oxidase deficiency

## Abstract

Dysferlinopathies are caused by mutations in the *DYSF* gene and patients may present with proximal or distal myopathy. Dysferlin is responsible for membrane resealing, and mutations may result in a defect in membrane repair following mechanical or chemical stress, causing an influx of Ca^2+^. Since mitochondria are involved in Ca^2+^ buffering, we hypothesised that mitochondrial defects may be present in skeletal muscle biopsies from patients with mutations in this gene. The aim was to characterise mitochondrial defects in muscle from patients with dysferlinopathies. Here, we analysed skeletal muscle biopsies for eight patients by quadruple immunofluorescent assay to assess oxidative phosphorylation protein abundance. Long-range PCR in single muscle fibres was used to look for presence of clonally expanded large-scale mitochondrial DNA rearrangements in patients' skeletal muscle (n = 3). Immunofluorescence demonstrated that the percentage of complex I- and complex IV-deficient fibres was higher in patients with *DYSF* mutations than in age-matched controls. No clonally expanded mtDNA deletions were detected using long-range PCR in any of the analysed muscle fibres. We conclude that complex I and complex IV deficiency is higher in patients than age matched controls but patients do not have rearrangements of the mtDNA. We hypothesise that respiratory chain deficiency may be the results of an increased cytosolic Ca^2+^ concentration (due to a membrane resealing defect) causing mitochondrial aberrations.

## Highlights

• Complex I deficiency is higher in patients with DYSF mutations than controls.
• Complex IV deficiency is higher in patients with DYSF mutations than controls.
• DYSF mutations may alter Ca^++^ buffering causing respiratory chain deficiency.
• No evidence of mitochondrial DNA deletions detected in dysferlin patients.

## 1 Introduction

Dysferlin mutations cause a number of different phenotypes including limb girdle muscle dystrophy type 2B (LGMD2B), Miyoshi distal myopathy (MM) [1], [2] and distal myopathy with Anterior Tibialis onset (DMAT) [3]. Links between mutation type, location and phenotype are not straight forward [4], [5]. These conditions demonstrate a multitude of dystrophic features including necrotic fibres, fibre splitting, variation in fibre size, and sometimes inflammatory infiltrates [6]. Dysferlin is responsible for plasma membrane repair, vesicle fusion and membrane trafficking. The ferlin protein group to which dysferlin belongs all have Ca^2+^ sensitive C2 domains [7]; dysferlin itself has seven C2 domains with variable affinities for Ca^2+^ [8]. It has previously been demonstrated that an influx of Ca^2+^ through the site of membrane injury triggers dysferlin-mediated membrane repair [9]. Patients with mutations in the dysferlin gene often have impaired membrane resealing following mechanical or chemical stress, causing an influx of Ca^2+^. This is particularly relevant in muscle where mechanical stress due to muscle contraction is quite common and Ca^2+^ regulation is highly important.

To date, two reports have been published documenting mitochondrial abnormalities in patients with dysferlin mutations. The first report demonstrated accumulation of subsarcolemmal mitochondria in some patients' muscle fibres including one patient with ragged red fibres and paracrystalline mitochondrial inclusions [10]. More recently up to 10% of muscle fibres were reported to be cytochrome *c* oxidase (COX or complex IV) deficient, and some of these fibres have increased mtDNA copy number [11]. In the same study, a reduced complex IV and complex III levels were observed in some patients, while others showed a significant downregulation of complex I and complex IV activities and only a mild reduction of complex III.

Here, we employ a recently developed immunofluorescent technique [12] to measure levels of complex I and complex IV normalised to porin as a mitochondrial mass marker. This technique, unlike COX/SDH histochemistry allows us to accurately and objectively quantify the extent of mitochondrial respiratory chain deficiency. We also hypothesise that increased mitochondrial fission due to increased cytosolic Ca^2+^ may reduce mtDNA stability. Therefore, we also employ long-range PCR to look for the presence of large-scale mtDNA rearrangements. Accurate and objective quantification of complex I and complex IV levels and mtDNA deletion analysis provide a novel addition to previous investigations in patients with dysferlin mutations.

## 2 Patients and methods

### 2.1 Patient cohort

Muscle biopsies were taken for diagnostic purposes from patients (n = 8), with a suspected neuromuscular condition; all patients consented to the use of the sample for research. Diagnostic immunohistochemistry and western blotting showed an absence of dysferlin. Sequencing of the *DYSF* gene identified pathogenic mutations (Table 1), confirming a diagnosis of dysferlinopathy. Eight patients included in the study (numbered 1–8 based on age); six were diagnosed with Limb Girdle Muscular Dystrophy type 2B (LGMD2B) and two with Miyoshi myopathy (MM). Patients were numbered 1 to 8 based on age at biopsy. A muscle biopsy from a previously-reported patient with an mtDNA maintenance disorder and multiple deletions due to recessive *RRM2B* variants [13] was used as a positive control, as well as 3 healthy control biopsies from subjects aged 18, 35 and 52 years at the time of biopsy.

### 2.2 Histochemistry

Cryosections (10 µm) were obtained from transversely-orientated muscle blocks and subjected to COX/SDH (cytochrome c oxidase/succinate dehydrogenase) histochemistry. Three serial sections were used to detect: COX activity, SDH activity and combined COX/SDH activity, as described previously [14]. Numbers of COX-deficient fibres were counted in a defined area for each section using the meander scan function in Stereo Investigator and a Stereology microscope (Olympus BX51). Serial sections were stained with haematoxylin and eosin (H&E) to examine muscle morphology. Slides were assessed independently by two investigators.

### 2.3 Quadruple immunofluorescence

A recently developed immunofluorescent assay was used to assess levels of complex I and complex IV relative to mitochondrial mass [12]. Images were stitched using ZEN (blue edition) before analysis in IMARIS (v.8) software, and statistical analysis was completed as described previously [12].

### 2.4 Laser microdissection and cell lysis

Three serial cryosections were cut. The first and last tissue sections underwent sequential COX/SDH histochemistry and were used to assess the degree of COX deficiency. The second tissue section underwent SDH histochemistry only and was used for mtDNA analysis. Single muscle fibres were laser microdissected from the second tissue section using a PALM system (Zeiss). Fibres from the second tissue section were captured into 15 µl single cell lysis buffer (0.5% SDS, 10 mM EDTA, 5% proteinase K).

### 2.5 Long-range PCR

Two rounds of PCR were employed to screen isolated muscle fibres for large-scale mtDNA rearrangements using PrimeSTAR GXL (TaKaRa, Clonetech). The reaction was prepared as follows: 1 µl cell lysate, 1× PrimeSTAR GXL reaction buffer, 0.2 µM dNTPs, 0.2 µM forward and reverse primers and 0.625 unit polymerase, in a total volume of 50 µl. The first round of PCR amplified a 16,179 bp region using primers 2180F (nucleotides m.2180–2209) and 1789R (nucleotides m.1760–1789) and the second round reaction amplified a 16,029 bp region using primers 2330F (nucleotides m.2330–2359) and 1789R (nucleotides m.1760–1789) (NC_012920.1). Cycling conditions were: 35 cycles of 10 seconds at 98 °C and 11 minutes at 68 °C. PCR products were separated through a 0.7% agarose gel with a 1 Kb ladder used to size amplicons.

## 3 Results

### 3.1 COX/SDH histochemistry and haematoxylin and eosin

**Fig. 1:**
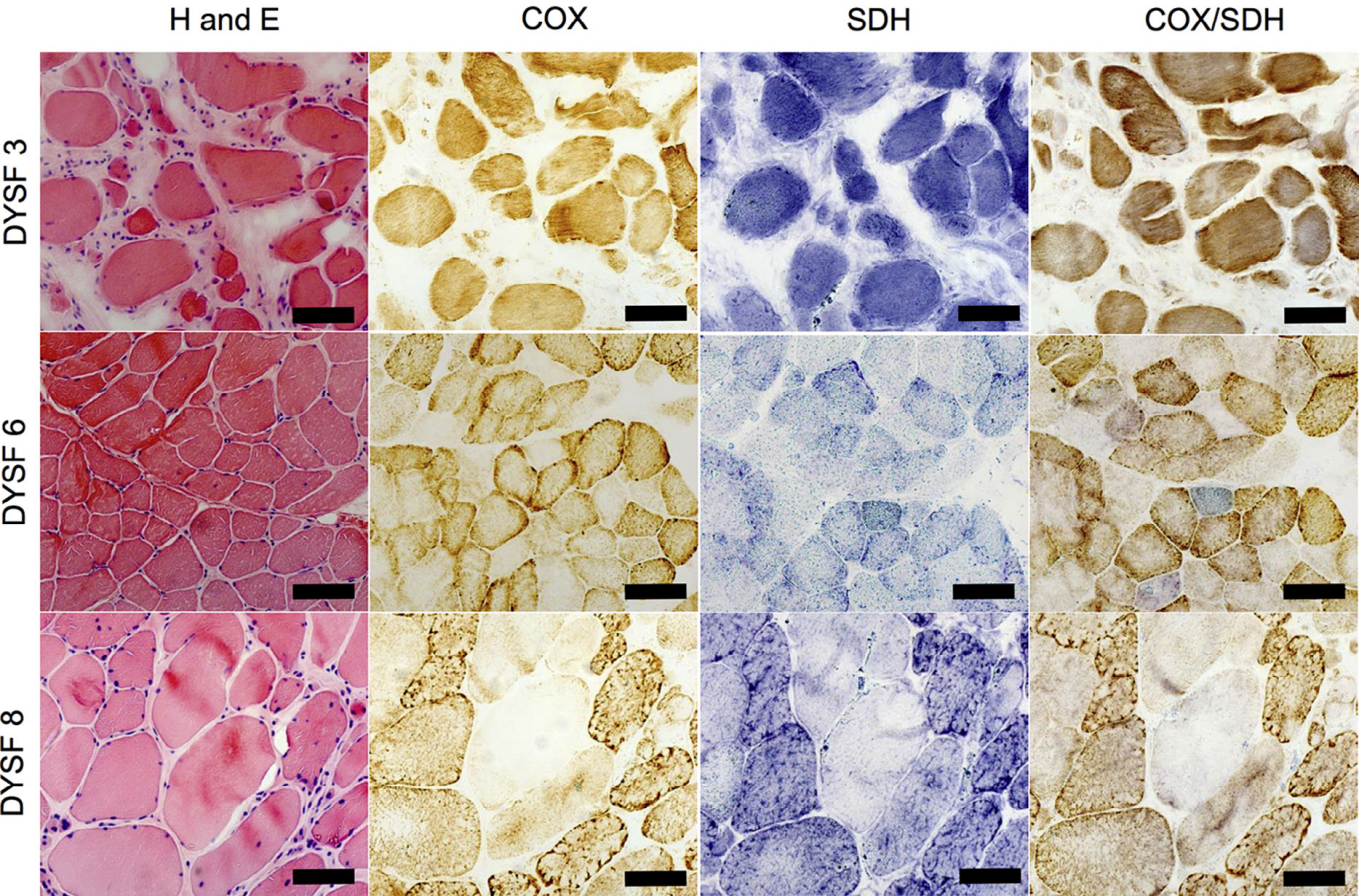
Histochemical analysis of serial muscle section. Notes: Serial muscle sections from patients 3, 6 and 8, for H&E, COX, SDH and sequential COX/SDH histochemistry. H&E demonstrates large variation in fibre size, with occasional internal nuclei and inflammation. Only DYSF 6 shows a clear cut COX negative fibre but all three biopsies show evidence of intermediate fibres.

H&E staining demonstrated a large variability in fibre size. There were also many necrotic fibres, internal nuclei and in some cases inflammatory infiltrates mostly associated with (but not limited to) necrotic fibres (Fig. 1, left column). COX/SDH histochemistry demonstrated a small number (typically less than 1%) of COX deficient fibres (Fig. 1, right column). However, many biopsies had a large number of fibres that appeared to have intermediate levels of COX deficiency, which was difficult to assess visually (Fig. 1, DYSF 3). Stereoinvestigator and a stereology microscope were used to count the number of COX positive, intermediate positive, intermediate negative and negative fibres in a defined region (Table 2). Due to very few truly COX-deficient fibres and many more COX-intermediate muscle fibres, deficiency level was likely to be under- or overestimated. Furthermore, counts between 2 investigators varied due to a large number of fibres intermediate for COX activity, which demonstrated the necessity of applying the objective quantitative immunofluorescence to more accurately assess COX levels.

### 3.2 Immunofluorescent analysis of respiratory chain complexes

**Fig. 2:**
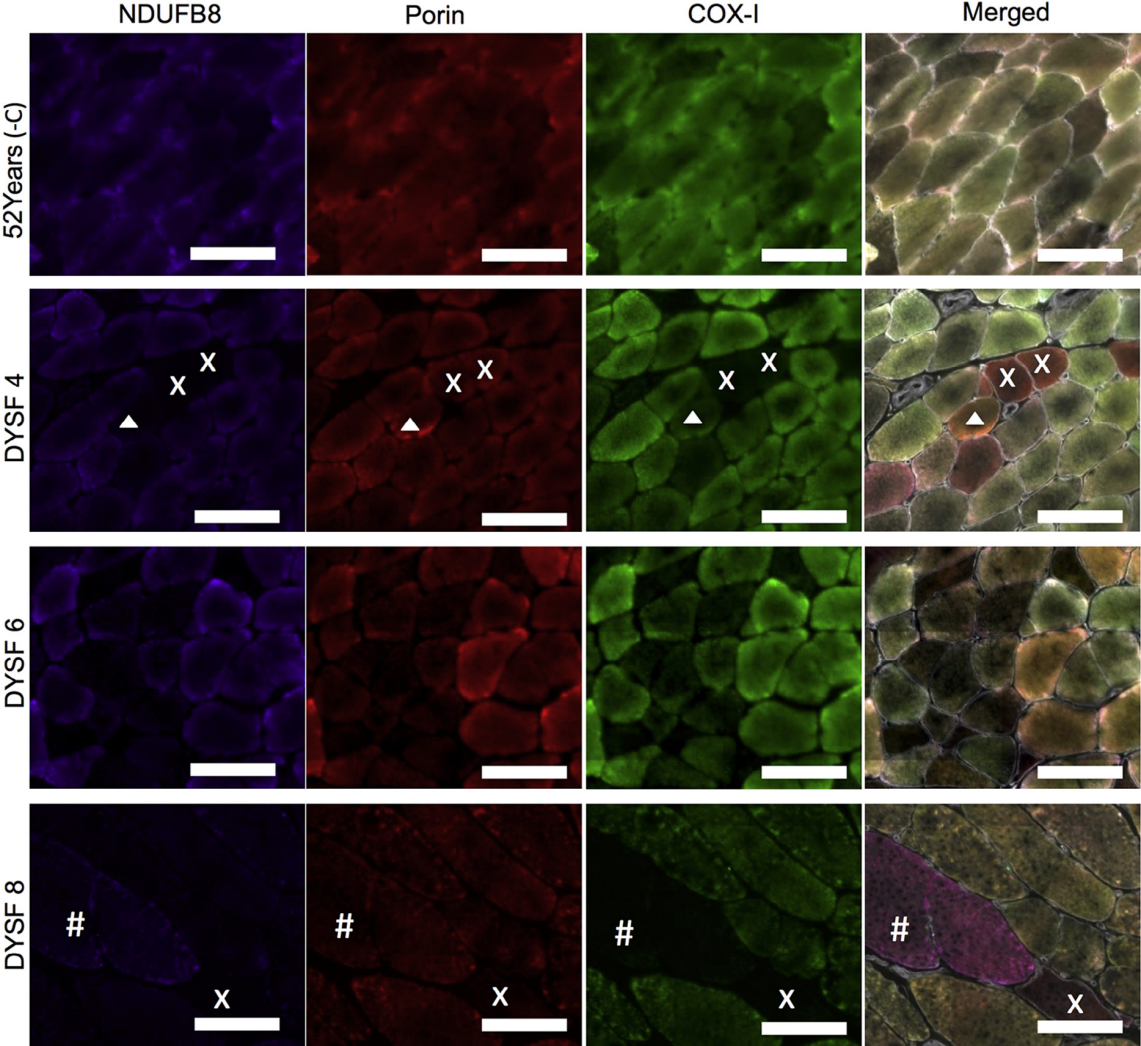
Quadruple immunofluorescent analysis of mitochondrial respiratory chain deficiency. Notes: Representative images of NDUFB8 (complex I) (purple), porin (red) and COX-I (complex IV) (red). Immunofluorescent staining in a 52 year old negative control and dysferlin patients 4, 6 and 8. Fibres deficient for complex I and complex IV (x in DYSF4 and DYSF8), complex IV only (# in DYSF8) and complex I only (arrow head in DYSF8) can be seen. Scale bar 100 µm.

Quadruple immunofluorescence allowed objective quantification of complex I and complex IV protein abundance. Results are summarised in Table 3. Immunofluorescent images for DYSF 4, 6 and 8 (Fig. 2) demonstrate complex I- and complex IV-deficient fibres. In 18 and 34 year old control biopsies, complex I deficiency is absent, but present in approximately 0.5% fibres in the 52 year old control. In contrast, complex I deficiency ranged from 0% in two patients to 29.4% in patient 3. These levels were much higher than we expected in age matched healthy individuals but lower than those found in the patient with recessive *RRM2B* variants (36.2%).

The 18 and 34 year old control biopsies demonstrated no complex IV deficient fibres and the 52 year old had approximately 0.5%. In contrast to this, 0.1–11.1% of patients' muscle fibres had evidence of complex IV deficiency. A higher proportion of muscle fibres are complex IV deficient than in the age-matched controls and previous reports of age-related complex IV deficiency [15]. However, in comparison to the patient with *RRM2B* variants whose biopsy harboured 23.2% complex IV deficient fibres, the degree of deficiency in the dysferlin patient is low.

**Fig. 3:**
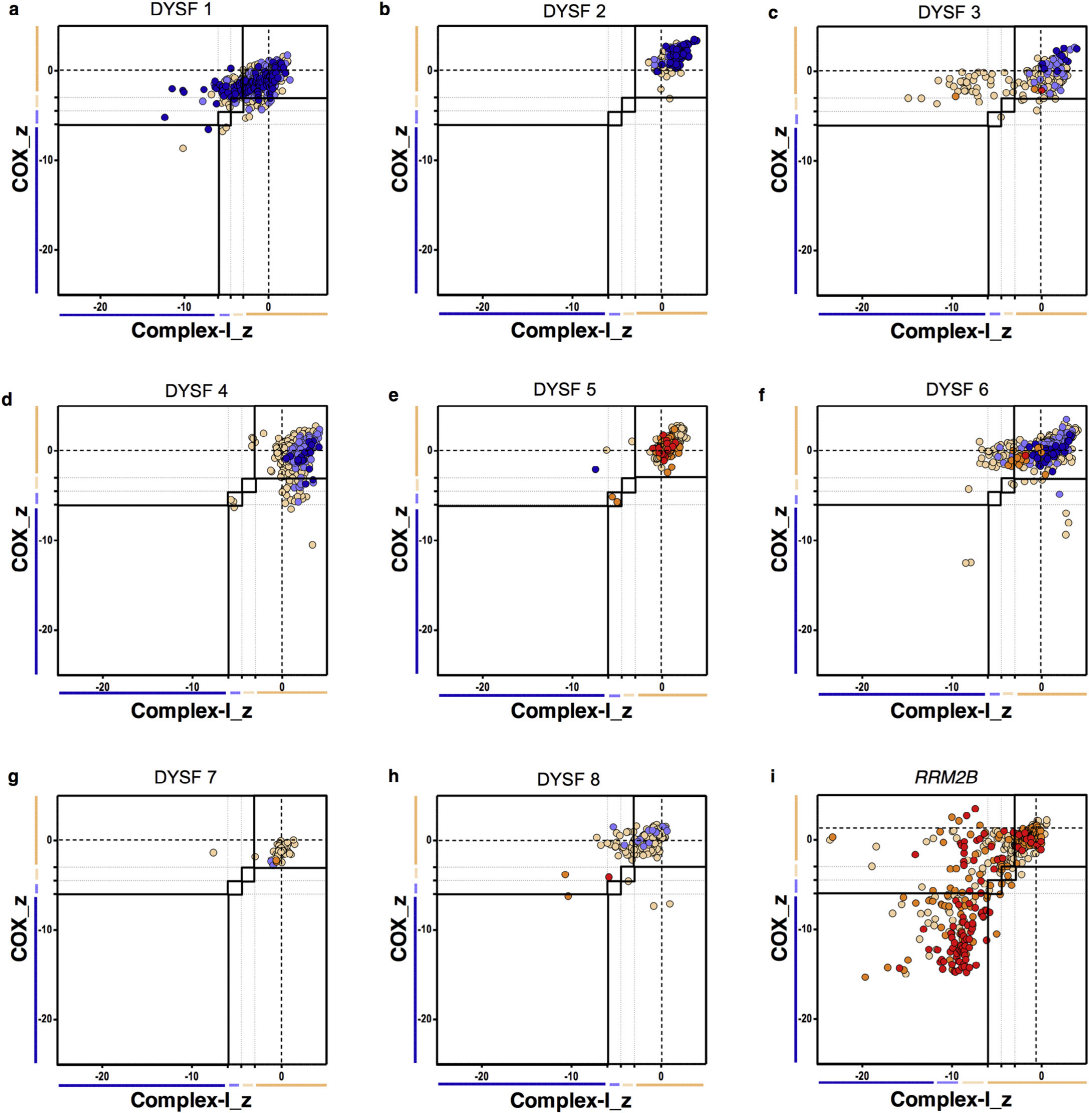
Mitochondrial respiratory chain expression profiles displaying complex I, complex IV and porin levels in patients with *DYSF* mutations and an *RRM2B* mutation as a mitochondrial disease control. (a) DYSF 1, (b) DYSF 2, (c) DYSF 3, (d) DYSF 4, (e) DYSF 5, (f) DYSF 6, (g) DYSF 7, (h) DYSF 8 and (i) RRM2B patient positive control. Notes: Each point gives the complex I and complex IV z-scores for a single fibre and is colour coded to indicate the porin category of the fibre (very low, dark blue; low, light blue; normal, beige; high, orange; or very high red). Bars next to the X and Y-axes indicate category of complex I or complex IV levels (dark blue, negative; light blue, intermediate negative; light beige, intermediate positive and beige, normal).

The respiratory expression profiles in Fig. 3 demonstrate three clear patterns of mitochondrial respiratory chain protein levels. Dysferlin patients 1, 3 and 6 (Fig. 3a, c and f respectively) had large number of fibres which were complex I-deficient. Dysferlin patient 4 (Fig. 3d) had a larger number of complex IV-deficient fibres than complex I-deficient fibres. Patients, 2, 5, 7 and 8 (Fig. 3b, e, g and h respectively) demonstrated very little deficiency affecting either respiratory chain complex. All dysferlinopathy patients had lower levels of respiratory chain deficiency than the *RRM2B* patient (Fig. 3i) but at 43 years of age, this disease control is older than the majority of dysferlin patients (many of whom are below the age of 35 years).

### 3.3 Mitochondrial DNA genetic analysis

**Fig. 4:**
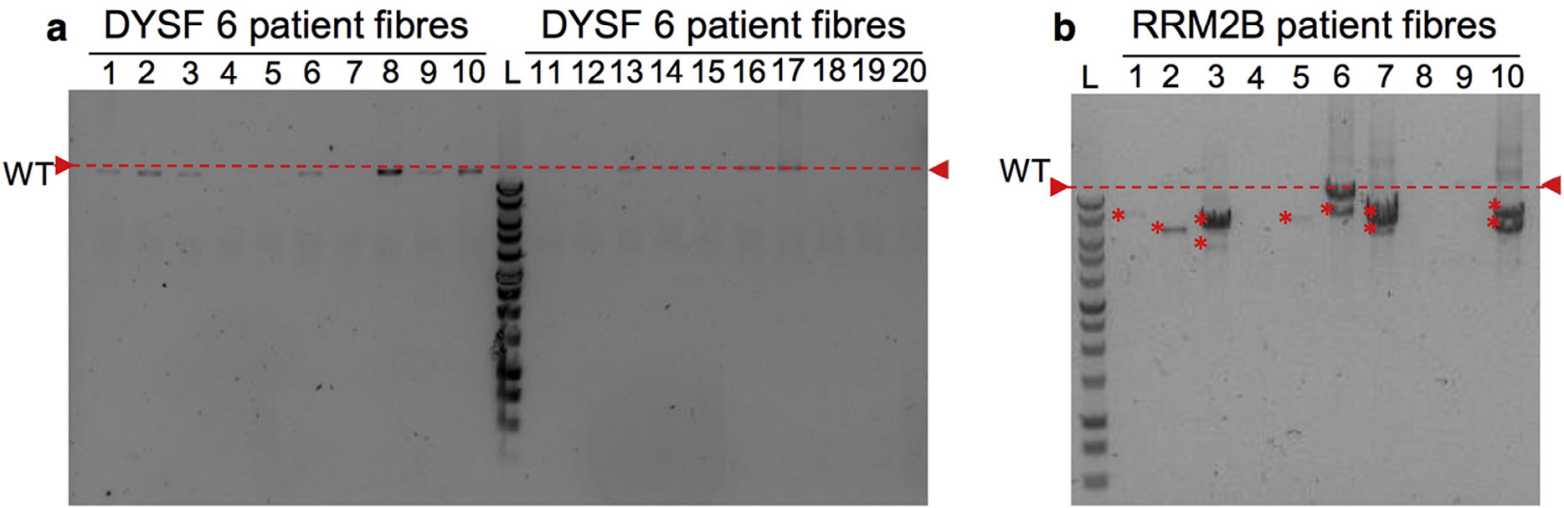
Long-range PCR analysis of mtDNA. Long-range analysis of dysferlin patient 6 (DYSF6) (representative). Notes: Red dashed line on all gels indicates wild type mitochondrial DNA size. 1Kb ladder (Promega) run on every agarose gel to size bands and a non-deleted, wild type DNA sample and a no template were included as controls. (a) Long-range PCR results for 20 single fibre lysates from DYSF 6 showed no deletions to be present, while (b) the *RRM2B* positive control long-range PCR results show multiple large-scale mtDNA deletions detected (*).

Patients 4, 6 and 8 were selected for genetic analysis, as these demonstrated the most COX-deficient fibres in sections subjected to COX/SDH histochemistry. Long-range PCR indicated a lack of deletions in all fibres for which a product was successfully amplified for each patient (Fig. 4a), while the patient harbouring compound *RRM2B* variants demonstrated multiple mtDNA deletions (Fig. 4b). In each of the dysferlin patients and an RRM2B patient, a small number of fibres failed to amplify.

## 4 Discussion

This study has demonstrated that the levels of complex IV (COX) and complex I deficiency are higher in some dysferlinopathy patients than in age-matched controls. The majority of deficient fibres were categorised as intermediate positive or intermediate negative rather than completely negative. Furthermore, the percentage of complex I-deficient fibres was higher than the percentage of complex IV deficient fibres in many of these patients.

Long-range PCR analysis of mtDNA from three patients, with high percentage of complex I and complex IV deficient fibres, demonstrated only full-length mtDNA amplicons. Despite an absence of large-scale mtDNA deletions, it is possible that the respiratory chain dysfunction may be due to deletions or duplications smaller than the resolution of the technique, or indeed mtDNA point mutations. As there is a greater percentage of complex I and complex IV deficiency in patients compared to controls, we hypothesise that mutations in the dysferlin protein which cause a defect in membrane resealing and alter the ability of dysferlin to maintain Ca^2+^ homeostasis may also have an impact on the mitochondria. If so, mitochondrial respiratory chain dysfunction could be secondary to the increased cytosolic calcium concentration which may affect mitochondrial topology.

Dysferlin, which has ferlin Ca^2+^ domains with variable affinities for Ca^2+^ helps to regulate cytosolic Ca^2+^ [10]. In the absence of functional dysferlin, cytosolic Ca^2+^ levels become abnormally high. Regulation of cytosolic Ca^2+^ levels is also a role of the mitochondria; as such, a shift in Ca^2+^ will affect their function. Ca^2+^ influx into the cytoplasm increases in mitochondrial fission and leads to fragmentation of the mitochondrial network [16]. Mitochondrial fusion is necessary for mtDNA stability and tolerance of mtDNA mutations, as it allows equilibration of the mtDNA maintenance machinery throughout the network and dilutes mutated mitochondrial DNA reducing the impact on mitochondrial function [17]. As such, mutations in the dysferlin gene that increase Ca^2+^ influx and impair muscle Ca^2+^ homeostatic ability of the muscle cause an increase in cytosolic Ca^2+^ concentration, fragmentation of the mitochondrial network and therefore allow mitochondrial dysfunction to accumulate and impact on tissue functionality.

Here, we demonstrate that complex I and complex IV deficiency is higher in patients with dysferlin mutations than in age-matched controls. We hypothesise that this may be due to interruption of calcium homeostasis because of the mutation in dysferlin and defect in membrane resealing. Further testing will be required to confirm this but may offer a potential therapeutic target.

## Figures and Tables

**Table 1:** Dysferlinopathy patient information.

Patient number	Gender	Age at onset (if known)	Age at biopsy	Mutation	CK	Phenotype
DYSF 1	F	10 (distal)	15.7	c.855+1delG; c.3031G>C, p.(Gly1011Arg)	3012	MM
DYSF 2	F	26	20.5	c.2434dup p.(His812Profs*53); c.1745G>T p.(Arg582Leu)	7586	LGMD2B
DYSF 3	F	26	21.9	Hom. c.2163-1G>T	13,000	LGMD2B
DYSF 4	M	Birth	24.7	Hom. c.1480+18T>C; c.4999G>A, p.(Glu1667Lys)	400–700	LGMD2B
DYSF 5	M	9	28.9	exon 5–10 deletion; c.757C>T, p.(Arg253Trp)	5000	LGMD2B
DYSF 6	M	Late teens (distal)	30.4	Hom. c.3059dupC, p.(Glu1021Glyfs*10)	20,000	MM
DYSF 7	F	N/K	47.1	c. 5509G>A, p.(Asp1837Asn); c.6124C>T, p.(Arg2042Cys)	5110	LGMD2B
DYSF 8	M	13	52.5	c.3512_3513delCT, p.(Ser1171Phefs*3); c.5908C>T, p.(Pro19070Ser)	6000	LGMD2B

Notes: Patients are organised in age order and all mutation nomenclature uses the primary transcript (NM_003494.3). All biopsies were taken from the quadriceps via needle biopsy.

N/K; Not known; M: Male; F: Female; Hom.: homozygous; LGMD2B: Limb girdle muscular dystrophy type 2B; MM: Miyoshi Myopathy.

**Table 2:** COX deficiency for dysferlin patients (n = 8) as assessed by sequential COX/SDH histochemistry.

Investigator	Patient	Total fibres	% COX positive	% Intermediate positive	% Intermediate negative	% COX negative
1	DYSF 1	1491	98.2%	1.8%	0.0%	0.0%
	DYSF 2	577	75.4%	18.4%	6.1%	0.1%
	DYSF 3	963	65.5%	21.9%	11.9%	0.6%
	DYSF 4	1774	97.7%	5.0%	0.1%	0.0%
	DYSF 5	397	86.9%	12.8%	0.3%	0.0%
	DYSF 6	563	80.8%	17.9%	0.9%	0.4%
	DYSF 7	132	69.7%	30.3%	0.0%	0.0%
	DYSF 8	453	92.5%	4.9%	2.0%	0.7%
2	DYSF 1	1357	83.6%	16.3%	0.07%	0.0%
	DYSF 2	382	71.7%	26.9%	1.5%	0.0%
	DYSF 3	328	65.9%	30.4%	3.7%	0.0%
	DYSF 4	1131	97.1%	2.6%	0.2%	0.1%
	DYSF 5	395	77.7%	22.0%	0.3%	0.0%
	DYSF 6	726	70.4%	27.1%	1.9%	0.6%
	DYSF 7	135	58.5%	31.1%	10.4%	0.0%
	DYSF 8	463	78.2%	20.3%	1.2%	0.4%

Notes: Fibres were classified as COX positive, COX intermediate positive, COX intermediate negative or negative in order to make it comparable with immunofluorescent assessment.

**Table 3:** Immunofluorescent analysis of respiratory chain protein expression.

Patient	Age/years	Complex I negative/%	Complex I deficient/%	Complex IV negative/%	Complex IV deficient/%
DYSF 1	15.7	2.03	12.26	*0.58*	6.62
18 years (-ve C)	18	0	0	*0*	0
DYSF 2	20.5	0	0.00	*0*	0.05
DYSF 3	21.9	26.1	29.42	*0*	11.05
DYSF 4	24.7	0.09	0.85	*1.13*	4.7
DYSF 5	28.9	0.15	0.48	*0.12*	0.33
DYSF 6	30.4	1.78	7.43	*1.24*	3.05
34 years (-ve C)	34	*0.18*	0.6	*0.18*	0.3
DYSF 7	47.1	0	0.00	*0*	5.05
52 years (-ve C)	52	*0*	0	*0*	0
DYSF 8	52.5	1.96	10.79	*0.44*	1.31

Notes: Summary of percentage of fibres classified as complex-I and complex-IV negative or deficient (negative and intermediate categories). Controls and patients are organised in age for easy comparison.
